# Exploring self-care practices, experiences, and interventions among adolescents and young adults with type 1 diabetes in Sub-Saharan Africa: a scoping review protocol

**DOI:** 10.1080/16549716.2026.2644032

**Published:** 2026-03-17

**Authors:** Dereje Wondim, Elena Keller, Hirut Abebe, Irén Tiberg

**Affiliations:** Department of Health Sciences, Faculty of Medicine, Lund University, Lund, Sweden

**Keywords:** Self-management, diabetes education, youth health, health services accessibility, scoping review methodology

## Abstract

This scoping review aims to systematically explore the extent, nature, and gaps in the existing literature on self-care practices among adolescents and young adults aged 10–24 years living with type 1 diabetes mellitus (T1D) in Sub-Saharan Africa. The review will examine self-care knowledge, attitudes, practices, perceived barriers and facilitators, and interventions developed to support self-care. Type 1 diabetes mellitus accounts for approximately 5–10% of all diabetes cases globally and disproportionately affects children and young people in low- and middle-income countries. Adolescence and young adulthood are critical developmental periods during which self-care behaviours are established and may influence long-term health outcomes. In Sub-Saharan Africa, adolescents and young adults with T1D face challenges including limited healthcare infrastructure, inadequate disease awareness, restricted access to insulin and diabetes supplies, and high out-of-pocket costs. This scoping review will include quantitative and qualitative studies, systematic reviews, and grey literature published in English from January 2004 onwards. The review will follow the Joanna Briggs Institute methodology for scoping reviews. A three-step search strategy will be applied across PubMed, MEDLINE, Web of Science, CINAHL, and Embase. Two independent reviewers will screen titles, abstracts, and full texts. Data will be extracted using a structured tool and synthesised using narrative, tabular, graphical, and thematic approaches.

## Paper Context

- **Main findings:** This protocol outlines a scoping review to map evidence on self-care among adolescents and young adults with type 1 diabetes in Sub-Saharan Africa.
- **Added knowledge:** The review will synthesise fragmented evidence and identify gaps in self-care knowledge, experiences, and interventions.
- **Global health impact for policy and action:** This scoping review will map existing evidence on self-care among adolescents and young adults with type 1 diabetes in sub-Saharan Africa, providing an overview of literature relevant to global health research and policy considerations.

## Background

Type 1 diabetes mellitus (T1D) is a chronic autoimmune condition characterised by immune-mediated destruction of insulin-producing beta cells in the pancreas, resulting in absolute insulin deficiency and lifelong dependence on exogenous insulin [1–3]. It is most commonly diagnosed in childhood and young adulthood and requires continuous self-care to prevent acute complications such as hypoglycaemia and diabetic ketoacidosis, as well as long-term complications including cardiovascular disease, nephropathy, neuropathy, and retinopathy [2–6]. Despite improvements in insulin therapy and diabetes care, T1D remains a condition without a cure or established preventive strategies [3,7]. In sub-Saharan Africa, the phenotype of type 1 diabetes is incompletely characterised, and accurate diagnosis is often complicated by atypical diabetes presentations and limited diagnostic resources [4]. Evidence suggests a later peak age of onset and a lower prevalence of islet autoantibodies among individuals diagnosed with type 1 diabetes in African populations compared with other regions [4].

Globally, T1D accounts for approximately 5–10% of all diabetes cases [5]. In Africa, an estimated 60,000 children were living with T1D in 2021, with nearly 20,000 new diagnoses reported that year [6]. Management of T1D in Sub-Saharan Africa remains particularly challenging due to delayed diagnosis, limited availability of insulin and glucose monitoring supplies, poor glycaemic control, and high treatment-related costs [6–8]. Studies from the region report high rates of acute and chronic complications and excess mortality among children and young people with T1D, reflecting structural inequities in access to care and continuity of treatment [9,10].

Although global knowledge of diabetes management has advanced over recent decades, effective self-care among individuals with T1D in Sub-Saharan Africa continues to be constrained by multiple systemic and contextual barriers [6–10]. These include inadequate healthcare infrastructure, shortages of trained healthcare professionals, limited diabetes education, and inequitable distribution of specialised services, which are often concentrated in urban tertiary facilities [11–13]. At the primary care level, community health workers may play an important role in diabetes follow-up but often lack specialised training and resources [13].

Adolescence and young adulthood represent critical periods for cognitive, emotional, and behavioural development. During these stages, the prefrontal cortex continues to mature, influencing decision-making, planning, and self-regulation, all of which are essential for effective diabetes self-care [14,15]. Establishing self-care routines between the ages of 10 and 25 years is particularly important, as behaviours formed during this period may have lasting implications for metabolic control and long-term health outcomes [15–17]. During early adolescence (10–12 years), diabetes self-care is typically embedded within a shared-care model involving caregivers and healthcare providers, with adolescents assuming increasing responsibility over time [16,17].

Improving self-care support is recognised as a key strategy for strengthening diabetes outcomes. The World Health Organization highlights self-management support as a cornerstone of effective chronic disease care [18]. Furthermore, children and adolescents living with T1D have the right to the highest attainable standard of health under Article 24 of the United Nations Convention on the Rights of the Child, yet many continue to experience substantial barriers to adequate and continuous care [19]. However, diabetes-related outcomes among adolescents remain suboptimal globally, including in low-resource settings [16]. Despite the growing burden of T1D in Sub-Saharan Africa, no comprehensive synthesis has mapped self-care practices, experiences, and interventions among adolescents and young adults in this region. The objective of this scoping review is to map and synthesise evidence on self-care practices, experiences, perceived barriers and facilitators, and interventions among adolescents and young adults aged 10–24 years living with type 1 diabetes mellitus in Sub-Saharan Africa.

## Methods

### Review questions

1. What is the extent, nature, and gaps in the existing literature on self-care practices and experiences among adolescents and young adults with T1D in Sub-Saharan Africa?
2. What is known about self-care knowledge, attitudes, practices, perceived barriers, and facilitators among adolescents and young adults with T1D in Sub-Saharan Africa?
3. What interventions have been developed or implemented to support self-care among adolescents and young adults with T1D in Sub-Saharan Africa, and what outcomes have been reported?

## Inclusion criteria

### Participants

Studies involving adolescents and young adults aged 10–24 years diagnosed with T1D will be included. This age range aligns with World Health Organization definitions of adolescents (10–19 years) and young adults (20–24 years). Studies including mixed-age populations will be included if analyses are reported separately for adolescents and/or young adults within the age range of interest, or if the reported mean age of participants falls within the 10–24-year range. Studies including both type 1 and type 2 diabetes will be included only when findings for T1D are reported separately.

### Concept

The review will focus on self-care practices and lived experiences related to T1D management, including knowledge, attitudes, behaviours, and engagement in activities such as insulin administration, blood glucose monitoring, dietary management, physical activity, and foot care. The review will also examine perceived barriers and facilitators to self-care, psychosocial experiences, support systems, and interventions designed to improve self-care. Given variability in diagnostic practices across Sub-Saharan Africa, T1D will be defined according to the criteria used in each included study.

### Context

The review will include studies conducted in Sub-Saharan Africa, as defined by the World Bank, across all settings including hospitals, primary care facilities, schools, communities, and households.

### Types of sources

Quantitative, qualitative, and mixed-methods studies will be included, along with systematic reviews, scoping reviews, and relevant grey literature such as policy documents and reports. Only studies published in English from January 2004 onwards will be included.

### Exclusion criteria

Studies focusing exclusively on adults aged 25 years or older, gestational diabetes, or diabetes types other than T1D without separate reporting will be excluded. Studies conducted outside Sub-Saharan Africa will also be excluded.

### Search strategy

The review will follow the Joanna Briggs Institute three-step search strategy. An initial limited search was conducted in PubMed, MEDLINE, and Web of Science to identify relevant keywords and index terms. A comprehensive search will subsequently be conducted in PubMed, MEDLINE, Web of Science, CINAHL, and Embase. Reference lists of included studies will be screened for additional sources. The full search strategy will be reported in Appendix A.

### Study selection

**Figure 1.**
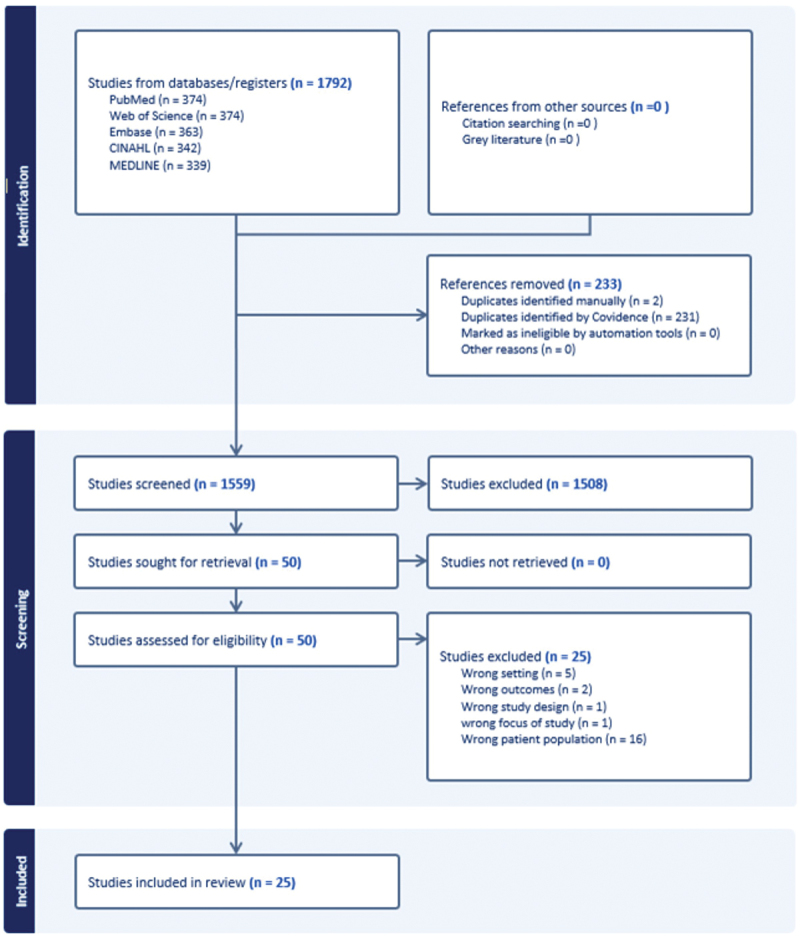
Prisma flowchart.

All identified citations will be imported into Covidence and duplicates removed. Two independent reviewers will screen titles and abstracts, followed by full-text screening. Disagreements will be resolved through discussion or consultation with a third reviewer. The study selection process will be reported using a PRISMA-ScR flow diagram (Figure 1) [20].

### Data extraction

Data will be extracted independently by three reviewers using a structured data extraction tool (Appendix B). Extracted data will include study characteristics, participant details, self-care knowledge, attitudes, practices, perceived barriers and facilitators, intervention characteristics, and outcomes. The extraction tool will be piloted and refined as necessary. All extracted data will be managed and stored within the Covidence systematic review software. Access to the data will be restricted to members of the review team directly involved in the study, and data will not be shared outside the research team.

### Data analysis and presentation

Findings will be synthesised using descriptive numerical summaries and qualitative thematic analysis. Results will be presented using tables, figures, and narrative summaries to map the evidence and identify knowledge gaps across settings, populations, and contexts.

## Discussion

Limiting inclusion to English-language publications may result in exclusion of relevant studies published in other languages commonly used in Sub-Saharan Africa, such as French or Portuguese. As a scoping review, this study will not assess the methodological quality of included sources.

## Conclusion

This scoping review will map existing evidence on self-care practices, experiences, and interventions among adolescents and young adults with T1D in Sub-Saharan Africa, providing an overview of literature relevant to global health research and policy considerations.

## Supplementary Material

PRISMA_Checklist.docx

## Appendix A. Search strategy

### PubMed

***Date searched: 18 February 2025***

**Table:**

Search number	Search	Retrieved
*#1*	*(diabetes mellitus[MeSH Terms]) OR (Diabet*[Title/Abstract]))) OR (diabetes mellitus[Title/Abstract])*	***689,428***
#2	*(self management[MeSH Terms]) OR (self care[MeSH Terms])) OR (patient education[MeSH Terms])) OR (health education[MeSH Terms])) OR (health knowledge, attitudes, practice[MeSH Terms])) OR (self efficacy[MeSH Terms])) OR (blood glucose self monitoring[MeSH Terms])) OR (adherence, medication[MeSH Terms])) OR (self administration[MeSH Terms])) OR (acceptability of health care[MeSH Terms])) OR (availability of health services[MeSH Terms])) OR (self monitoring[Title/Abstract])) OR (patient centered[Title/Abstract]))) OR (follow up[Title/Abstract])*	**1537326**
#3	*(adolescent[MeSH Terms]) OR (adolescence[MeSH Terms]) OR (youth[MeSH Terms]) OR (young adult[MeSH Terms]) OR (young people[Title/Abstract]) OR (adult children[MeSH Terms]) OR (adult, young[MeSH Terms])*	**1802222**
#4	*(subsaharan africa[MeSH Terms]) OR (africa south of the sahara[MeSH Terms]) OR (‘Angola’ OR ‘Benin’ OR ‘Botswana’ OR ‘Burkina Faso’ OR ‘Burundi’ OR ‘Cape Verde’ OR ‘Cameroon’ OR ‘Central African Republic’ OR ‘Chad’ OR ‘Comoros’ OR ‘Democratic Republic of the Congo’ OR ‘Republic of the Congo’ OR ‘Djibouti’ OR ‘Equatorial Guinea’ OR ‘Eritrea’ OR ‘Eswatini’ OR ‘Ethiopia’ OR ‘Gabon’ OR ‘Gambia’ OR ‘Ghana’ OR ‘Guinea’ OR ‘Guinea-Bissau’ OR ‘Ivory Coast’ OR ‘Kenya’ OR ‘Lesotho’ OR ‘Liberia’ OR ‘Madagascar’ OR ‘Malawi’ OR ‘Mali’ OR ‘Mauritania’ OR ‘Mauritius’ OR ‘Mozambique’ OR ‘Namibia’ OR ‘Niger’ OR ‘Nigeria’ OR ‘Rwanda’ OR ‘São Tomé and Príncipe’ OR ‘Senegal’ OR ‘Seychelles’ OR ‘Sierra Leone’ OR ‘Somalia’ OR ‘South Africa’ OR ‘South Sudan’ OR ‘Sudan’ OR ‘Tanzania’ OR ‘Togo’ OR ‘Uganda’ OR ‘Zambia’ OR ‘Zimbabwe’) OR (subsaharan[Title/Abstract])*	**480387**
#5	***#1 AND #2 AND #3 AND #4***	**403**

*(Each search limited to 2004 to current)*.

#### MEDLINE

**Table:**

Search number	Search	Retrieved
#1	***TX (‘Type 1 diabetes’ OR ‘type 1 diabet*’ OR ‘Types 1 diabetes mellitus’ OR ‘childhood diabetes’ OR ‘juvenile diabetes’ OR ‘T1DM’)***	***404,003***
#2	***TX (‘Self-Care’ OR ‘Self-Management’ OR ‘Patient Education’ OR ‘Health Education’)***	***326,389***
#3	***TX (‘adolescents’OR‘youth’OR‘children’)***	***3,945,445***
#4	*TX (‘Sub-Saharan Africa’ OR ‘Angola’ OR ‘Benin’ OR ‘Botswana’ OR ‘Burkina Faso’ OR ‘Burundi’ OR ‘Cape Verde’ OR ‘Cameroon’ OR ‘Central African Republic’ OR ‘Chad’ OR ‘Comoros’ OR ‘Democratic Republic of the Congo’ OR ‘Republic of the Congo’ OR ‘Djibouti’ OR ‘Equatorial Guinea’ OR ‘Eritrea’ OR ‘Eswatini’ OR ‘Ethiopia’ OR ‘Gabon’ OR ‘Gambia’ OR ‘Ghana’ OR ‘Guinea’ OR ‘Guinea-Bissau’ OR ‘Ivory Coast’ OR ‘Kenya’ OR ‘Lesotho’ OR ‘Liberia’ OR ‘Madagascar’ OR ‘Malawi’ OR ‘Mali’ OR ‘Mauritania’ OR ‘Mauritius’ OR ‘Mozambique’ OR ‘Namibia’ OR ‘Niger’ OR ‘Nigeria’ OR ‘Rwanda’ OR ‘São Tomé and Príncipe’ OR ‘Senegal’ OR ‘Seychelles’ OR ‘Sierra Leone’ OR ‘Somalia’ OR ‘South Africa’ OR ‘South Sudan’ OR ‘Sudan’ OR ‘Tanzania’ OR ‘Togo’ OR ‘Uganda’ OR ‘Zambia’ OR ‘Zimbabwe’)*	***857,994***
#5	***#1 AND #2 AND #3 AND #4***	***430***

*(Each search limited to 2004 to current)*.

### Appendix B. Data extraction instrument

**Table:**

**STUDY CHARACTERISTICS**
Study ID
Title
Lead author contact details
Country in which the study was conducted
Study setting (Facility)
Study setting (Geographical)
Methods – Aim of study
Study design
Year of publication
**POPULATION DETAILS**
Sample size
Age range (Mean age)
Gender distribution (Ratio)
Method of recruitment of participants
Total number of participants
INCLUSION CRITERIA
Population
Concepts
Context
Study participant included
**SELF-CARE PRACTICES INVESTIGATED**
Blood glucose monitoring
Insulin injection
Dietary management
Physical activities
Foot care practices
self-care knowledge
Self-care attitude
Other self-care practices
**EXPERIENCES AND CHALLENGES**
Positive experiences
Negative experiences
**BARRIERS AND FACILITATORS OF SELF-CARE PRACTICES**
Barriers
Facilitators
INTERVENTION DETAILS
Types of intervention
Outcome of the intervention
Barriers to the intervention
Facilitators of the intervention
**OTHER RELEVANT FINDINGS OF THE STUDY**
Findings
Gaps Identified
Suggestions for Future Research
